# Salivary Antigen SP32 Is the Immunodominant Target of the Antibody Response to *Phlebotomus papatasi* Bites in Humans

**DOI:** 10.1371/journal.pntd.0001911

**Published:** 2012-11-29

**Authors:** Soumaya Marzouki, Maha Abdeladhim, Chaouki Ben Abdessalem, Fabiano Oliveira, Beya Ferjani, Dana Gilmore, Hechmi Louzir, Jesus G. Valenzuela, Mélika Ben Ahmed

**Affiliations:** 1 Laboratory of Transmission, Control and Immunobiology of Infection, LR11IPT02, Institut Pasteur de Tunis, Tunis, Tunisie; 2 Vector Molecular Biology Section, Laboratory of Malaria and Vector Research, National Institute of Allergy and Infectious Diseases, National Institutes of Health, Rockville, Maryland, United States of America; 3 Faculté de Médecine de Tunis, Université Tunis El Manar, Tunis, Tunisie; Lancaster University, United Kingdom

## Abstract

**Background:**

Zoonotic cutaneous leishmaniasis (ZCL) due to *Leishmania major* is highly prevalent in Tunisia and is transmitted by a hematophagous vector *Phlebotomus papatasi* (*P. papatasi*). While probing for a blood meal, the sand fly injects saliva into the host's skin, which contains a variety of compounds that are highly immunogenic. We recently showed that the presence of anti-saliva antibodies was associated with an enhanced risk for leishmaniasis and identified the immunodominant salivary protein of *Phlebotomus papatasi* as a protein of approximately 30 kDa.

**Methodology/Principal Findings:**

We cloned and expressed in mammalian cells two salivary proteins PpSP30 and PpSP32 with predicted molecular weights close to 30 kDa from the Tunisian strain of *P. papatasi*. The two recombinant salivary proteins were purified by two-step HPLC (High-Performance Liquid Chromatography) and tested if these proteins correspond to the immunodominant antigen of 30 kDa previously shown to be recognized by human sera from endemic areas for ZCL and exposed naturally to *P. papatasi* bites. While recombinant PpSP30 (rPpSP30) was poorly recognized by human sera from endemic areas for ZCL, rPpSP32 was strongly recognized by the tested sera. The binding of human IgG antibodies to native PpSP32 was inhibited by the addition of rPpSP32. Consistently, experiments in mice showed that PpSP32 induced the highest levels of antibodies compared to other *P. papatasi* salivary molecules while PpSP30 did not induce any detectable levels of antibodies.

**Conclusions:**

Our findings demonstrate that PpSP32 is the immunodominant target of the antibody response to *P. papatasi* saliva. They also indicate that the recombinant form of PpSP32 is similar to the native one and represents a good candidate for large scale testing of human exposure to *P. papatasi* bites and perhaps for assessing the risk of contracting the disease.

## Author Summary

*Leishmania* is transmitted by female sand flies and deposited during a blood meal together with saliva. Saliva contains a vast repertoire of pharmacologically active molecules that facilitate the acquisition of the blood meal and contribute to the establishment of the infection. These molecules can induce the production of anti-saliva antibodies, which can be used as markers of exposure to the vector bite. Epidemiological studies using sand fly salivary gland extract as antigens are hampered by the difficulty in obtaining large amounts of salivary glands. In the present study, we have investigated the use of recombinant salivary proteins from the Tunisian strain of *Phlebotomus papatasi*, the main vector of zoonotic cutaneous leishmaniasis (ZCL) in Tunisia, as an alternative method for screening of exposure to the sand fly bites. We primarily identified PpSP32 as the immunodominant protein and described the low level of polymorphisms in the amino acid sequence of the target protein. Further, we tested the suitability of using the recombinant form of this protein to estimate positive anti-saliva antibodies in serum samples of individuals from an endemic area for ZCL. Our findings indicate that this protein represents a promising epidemiological tool that can aid in implementing control measures against leishmaniasis.

## Introduction

Zoonotic cutaneous leishmaniasis (ZCL) is highly prevalent in Central Tunisia where it is a public health problem with an annual incidence of ∼5,000 cases [1]. The etiological agent is an Old World *Leishmania* species, *Leishmania major*, which is transmitted by the sand fly vector, *Phlebotomus papatasi*. Several works showed that either humans or animals exposed to sand fly bites or experimentally immunized with saliva could develop antibodies that specifically recognize salivary proteins [2]–[6]. Identification of the sand fly salivary proteins targeted by the antibody response of mammalian hosts would increase our understanding of vector-host interactions and could also help in developing new epidemiological tools to correlate host exposure to vector sand flies with susceptibility to leishmaniasis. A relationship between the level of specific antibodies to saliva, vector exposure and risk of contracting disease has been, indeed, demonstrated for different vector-host models including leishmaniasis [4], [7]–[12].

We have recently reported that 83% of 200 donors living in areas of *Leishmania major* transmission in Tunisia produced anti-saliva IgG antibodies, primarily of the IgG4 isotype. The median level of the anti-saliva antibodies was significantly greater in patients who later developed ZCL compared to donors who did not, suggesting that the presence of such specific antibodies is associated with an enhanced risk factor of triggering the disease [13]. We additionally showed that positive sera reacted differentially with seven different salivary proteins and that a protein of approximately 30 kDa was prominently recognized by all the human sera tested.

Herein, we primarily identify PpSP32 as the immunodominant protein and show its low level of polymorphism in the sequence of the target protein when compared to the homologue protein from a different geographical area (Middle East). We further demonstrated the suitability of using the recombinant form of this protein to estimate positive anti-saliva antibodies in serum samples of individuals living in endemic areas of ZCL.

## Methods

### Ethics statement

All experiments were conducted according to the principles expressed in the Declaration of Helsinki. The study was approved by the ethic committee of the Institute Pasteur of Tunis. All parents/guardians provided consent on behalf of all child participants and provided written informed consent for the collection of blood samples and subsequent analyses. All animal procedures were reviewed and approved by the National Institute of Allergy and Infectious Diseases (NIAID) Animal Care and Use Committee and handled in accordance to the Guide for the Care and Use of Laboratory Animals and with the NIH OACU ARAC guidelines.

### Study population and samples

Peripheral blood samples were collected from 66 children (age ranging from 7 to 10 years with a median of 8.5 years) living in central regions of Tunisia (El Guettar and Souk Ejjdid). These regions are endemic for ZCL caused by *Leishmania major* and characterized by the presence of *P. papatasi* at high frequencies [14]. All donors were part of a previous study of ZCL in Tunisia [13]. For some experiments, peripheral blood samples were collected from 20 randomly selected donors (median age of 32 years) living in North regions of Tunisia (Utique and Menzel Bourguiba) characterized by the presence of *P. perniciosus* and *P. perfiliewi* and the absence of *P. papatasi* and from 20 randomly selected individuals (median age of 36 years) living in other Central regions of Tunisia (Kairoun) characterized by coexistence of *P. perniciosus* and *P. papatasi* [14].

### Salivary glands from Tunisian *P. papatasi*

Sand fly salivary glands, provided by Pr. E. Zhioua (Pasteur Institute of Tunis), were from a colony of *P. papatasi* that originated from El Felta, an endemic focus of ZCL located in the governorate of Sidi Bouzid in Central Tunisia (North Africa) [14]. The glands were dissected out in phosphate saline buffer using pliers and disrupted by 3 freezing and thawing cycles. After centrifugation, the supernatants were stored at −80°C with 10% glycerol.

### Cloning and sequencing of Tunisian PpSP30 and PpSP32

Salivary glands of 1 to 2-day-old females were dissected in phosphate saline buffer (Invitrogen) as previously described [13] and stored in RNA later (Qiagen, Hilden, Germany). Total RNA extraction was performed using RNeasy Mini Kit (Qiagen). The extracted RNA was then reverse transcribed using the Murine-Mooloney Leukemia Virus (MMLV) reverse transcriptase and random hexamers (Promega, Madison, WI, USA) according to standard procedure.

The cDNAs of PpSP30 and PpSP32 (from NH2 terminus to stop codon) were amplified from *P. papatasi* salivary protein cDNA by PCR using *Taq* polymerase (Invitrogen) and the following specific primers: Forward SP30 (5′- CAG TTT CAT CTT GCA AAA TG -3′); Reverse SP30 (5′- GGC TTT CCA ACA CAT TGT ATT TCA -3′); Forward SP32 (5′- ACA GCA TTT TGT GGA AAA TC-3′); Reverse SP32 (5′- CAG AAA AAA AGA ATA ACC TCA TG -3′). The PCR products were immediately cloned using TA cloning Kit (Invitrogen) following the manufacturer's specifications. The recombinant plasmid pCR 2.1-PpSP30 and pCR2.1-PpSP32 were purified from *Esherishia Coli* DH5α using Wisard Mini prep Kit (Promega) and then sequenced using specific primers as well as a vector specific primer (T7 Universal Primer; 5′-TAATACGACTCACTATAGGG-3′).

### *In silico* amino acid sequence analysis of the Tunisian PpSP30 and PpSP32 and the related GenBank proteins

The amino acid sequence as well as the structure of the Tunisian PpSP30 and PpSP32 were compared to the related GenBank sequences (PpSP30; GenBank ID: AF335489.1) and (PpSP32; GenBank ID: AF335490.1), using Clustal Wallis (http://www.ebi.ac.uk/Tools/msa/clustalw2/), Blast (http://blast.ncbi.nlm.nih.gov/), PredictProtein (www.predictprotein.org/) and bcepred (www.imtech.res.in/raghava/bcepred) softwares.

### Cloning of PpSP30 and PpSP32 into a VR2001-TOPO expression vector

DNA of the targeted molecule (PpSP30 or PpSP32) was amplified by polymerase chain reaction (PCR) using a forward primer deduced from the amino-terminus sequence (starting after the signal peptide) and a reverse primer encoding a hexahistidine motif. The following specific primers were used: Forward SP30 (5′- TGG CGA TTT CCT AGG AAT GGA GA -3′); His-Reverse SP30 (5′- TTA ATG ATG ATG ATG ATG ATG GTA TTT CCA AGA TTC AAT ATC A-3′); Forward SP32 (5′- GCA AGC ACA ATT CCC ATT CAG AGT -3′); His-Reverse SP32 (5′- TCA ATG ATG ATG ATG ATG ATG AGC CTT GAA AGT TTT GAT ACG TCC A -3′).

The PCR conditions were: one hold for 5 min at 94°C, two cycles of 30 s at 94°C, 1 min at 46°C, 1 min at 72°C and 23 cycles of 30 s at 94°C, 1 min at 52°C, 1 min at 72°C and one hold of 7 min at 72°C. The PCR product was cloned into the VR2001-TOPO vector as previously described [15] and then sequenced.

### Production and purification of recombinant PpSp30 and PpSp32 proteins (rPpSp30 and rPpSp32)

VR-2010 plasmid coding for the PpSP32 protein containing a 3′ histidine tag was sent to the Protein Expression Laboratory at NCI-Frederick (Frederick, Maryland) for expression in HEK-293F cells. The supernatant was collected after 72 hours and concentrated from 500 ml to 300 ml using a Stirred Ultrafiltration Cell unit (Millipore) with a 10 kDa ultrafiltration membrane (Millipore). The volume was returned to 1 L by the addition of 500 mM NaCL and 10 mM Tris, pH 8.0. The protein was purified by an HPLC system (DIONEX) using two 5 ml HiTrap Chelating HP columns (GE Healthcare) in tandem and charged with 0.1 M NiSO_4_. The protein was detected at 280 nm and eluted by an imidazole gradient as follows: 0–5 min, 100% Buffer A (20 mM NaH_2_PO_4_, 20 mM Na_2_HPO_4_, pH 7.4, 500 mM NaCl); 5–15 min, a gradient of 0% to 100% Buffer B (Buffer A+50 mM imidazole); 15–20 min, a gradient of 0% C (Buffer A+500 mM imidazole) to 10% C (90% B); 20–25 min, 90% B and 10% C; min 25–30, a gradient of 10% C to 20% C (80% B); 30–35 min, 80% B and 20% C; 35–40 min, a gradient of 20% C to 100% C; and 40–50 min, 100% C. Eluted proteins were collected every minute in a 96-well microtiter plate using a Foxy 200 fraction collector (Teledyne ISCO). Fractions corresponding to peak(s) were selected and run on a NuPage Bis-Tris 4–12% Gel (Novex) with MES running buffer under reducing conditions as per manufacturer's instructions. Briefly, NuPage LDS sample buffer (Invitrogen), NuPage reducing agent (Invitrogen), and sample were combined and heated to 70°C for 10 min. Samples were loaded in gel with SeeBlue Plus2 Pre-Stained Standard (Invitrogen) and run at 200 V for 35 min with an expected current of 100–125 mA (start), 60–80 mA (end). After run, gel was stained with Coomassie Blue (0.025%) to visualize proteins. Appropriate fractions as determined by molecular weight compared to standard in gel were pooled and concentrated to 1 ml using a 10 kDa Amicon Ultra Centrifugal Filter (Millipore). The protein sample was then injected into a g2000sw molecular sieving column (Tosoh Biosciences) with a 1 ml loop connected to HPLC (DIONEX) with phosphate buffer (PBS) pH 7.2 as the buffer for further purification. The protein was detected at 280 nm and the fractions were collected as described above. Appropriate fractions were determined as described above and pooled. Concentration was measured by using a NanoDrop ND-1000 spectrophometer at 280 nm and calculated using the extinction coefficient of the protein.

### Detection of human serum antibody anti-rPpSP30 and anti-rPpSP32 by ELISA

IgG antibodies against recombinant forms of PpSP30 and PpSP32 as well as against total salivary gland extract (SGE) were measured by ELISA (Enzyme-Linked Immunosorbent Assay). The wells (NUNC, Maxisorp, Roskilde, Denmark) were coated overnight with rPpSP30 or rPpSP32 (2 µg/ml = 0.1 µg/well) or SGE (0.5 glands per well) in 0.1 M carbonate-bicarbonate buffer (pH 9.6) at 4°C. The wells were then washed in PBS with 0.1% Tween 20 (PBS-T) and then incubated with 0.5% gelatin in PBS buffer with 0.1% Tween 20 for 1 hour at 37°C to block free binding sites. After washing, diluted sera (1∶200) were incubated for 2 hours at 37°C. Antibody-antigen complexes were detected using peroxidase-conjugated anti-human IgG antibody diluted at 1∶10000 (Sigma, St. Louis, MO, USA) for 1 hour at 37°C and visualized using 3,3′,5,5′Tetramethylbenzidine (TMB) (BD Biosciences, San Diego, CA, USA). The absorbance was measured using an automated ELISA reader (Awareness Technology Inc.) at 450 nm wavelength.

### Western blot analyses

Recombinant salivary proteins (0.7 to 1.6 µg per well) were run on a 15% SDS-PAGE. The separated proteins were then transferred onto a nitrocellulose membrane. The membrane was incubated 2 hours with a blocking buffer containing 5% non fat milk and then cut into strips; each strip was incubated overnight with diluted human sera at 1∶200 or 2 hours with monoclonal anti-polyhistidine antibody (Sigma) at 1∶2000. After washing, the strips were incubated with horseradish peroxidase-linked anti-human IgG antibody (Sigma) at 1∶10000 for 1 hour at room temperature. After five washings, positive bands were visualized using enhanced chemiluminescence (Amersham, Saclay, France). For this experiment, the RPN800E molecular weight marker (Amersham) was used.

For inhibition tests, human sera exhibiting IgG anti-SGE antibodies were mixed with the recombinant proteins (pre-adsorption step) at 10 µg/ml for 2 hours at room temperature. Salivary gland extracts (SGE) were separated on 15% SDS-PAGE and transferred onto a nitrocellulose membrane as previously described [13]. Each strip was then incubated overnight with mixture of positive serum + rPpSP30 and/or rPpSP32. The presence of IgG anti-SGE was revealed as previously described [13].

For detection of antibodies from animals immunized with DNA plasmids or salivary gland homogenate, western blot analysis was performed by electrophoresis of 30 pairs of *P. papatasi* salivary glands on a 4–20% Tris-glycine polyacrylamide gel. The salivary proteins were transferred to a nitrocellulose membrane and the membrane blocked with 5% (w/v) non-fat milk in Tris-buffered saline (TBS)-0.05% Tween, pH 8.0, overnight at 4°C. After washing with TBS-T, pH 8.0, the membrane was placed on a mini-protean II multiscreen apparatus (Bio-Rad, Hercules, CA), and different lanes were incubated with various sera (1∶100 dilution) for 2 hours at room temperature. After washing with TBS-T, pH 8.0, three times for 5 min, the membrane was incubated with alkaline phosphate-conjugated anti-mouse IgG (Promega) (1∶10,000) for 1 hr at room temperature. Membranes were developed by addition of the Western Blue stabilized substrate for alkaline phosphatase (Promega), and the reaction was stopped by washing the membrane with deionized water.

### Coomassie blue- and silver-stained SDS-PAGE

Recombinant PpSP32 (6 µg/well) was run on a 15% SDS- PAGE gel and the gel was then stained with Coomassie-blue. The RPN800E molecular weight marker (Amersham) was used. In one experiment, the different bands of rPpSP32 were cut from the gel and mass spectrometry was performed as previously described [13]. Recombinant PpSP30 (100 ng/well) was run on a 12% SDS- PAGE gel and the gel was then stained using the silverQuest Silver Staining Kit (Invitrogen) according to manufacture instructions. The SeeBlue Plus2 Pre-Stained standard (Invitrogen) was used.

### Immunization of mice with DNA encoding *P. papatasi* molecules

C57BL/6 mice from 4 to 6 weeks old were purchased from Charles River Laboratories Inc. Mice were kept under pathogen free conditions. DNA coding for the 10 most abundant *P. papatasi* salivary proteins were cloned into a VR2001-TOPO vector as described elsewhere [15]. Mice were immunized intradermally in the right ear, three times every two weeks with 500 ng/µl of DNA plasmid or half a pair of salivary glands diluted in 10 µl of sterile saline solution. Sera of mice obtained two weeks after the last immunization were tested for IgG antibody levels by ELISA, as described elsewhere [16].

### Statistical analysis

The correlation between the OD (optical density) of tested sera obtained in ELISA test with recombinant PpSP32 and total SGE was assessed using Spearman's rank correlation. In mouse experiments, statistical differences among multiple groups were analyzed using one-way ANOVA followed by Tukey's Multiple Comparison Test. Statistical significance was assigned to a value of p<0.05.

## Results

### Cloning and sequencing of PpSP30 and PpSP32 cDNA

**Figure 1:**
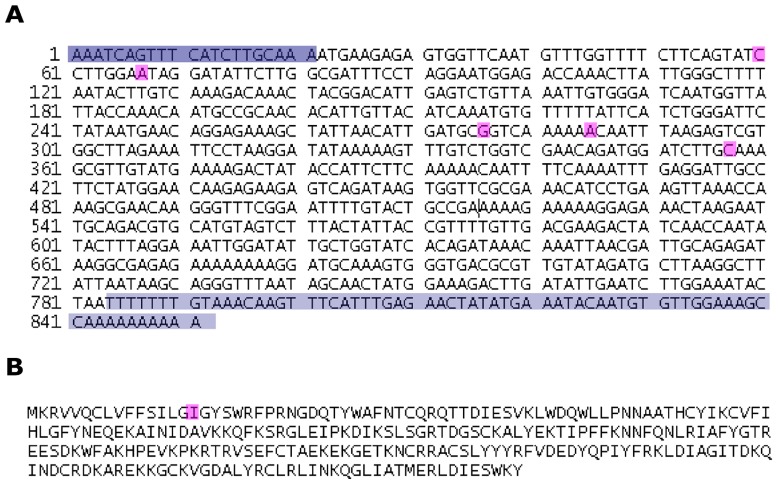
Sequences of the Tunisian *Phlebotomus papatasi* 30 kDa salivary protein. (**A**) mRNA sequence of PpSP30. (**B**) Protein sequence of PpSP30. Differences in the nucleotide and protein sequence PpSP30 described in the GenBank (GenBank AF335489.1) are highlighted in pink. Non coding sequence is highlighted in purple.

**Figure 2:**
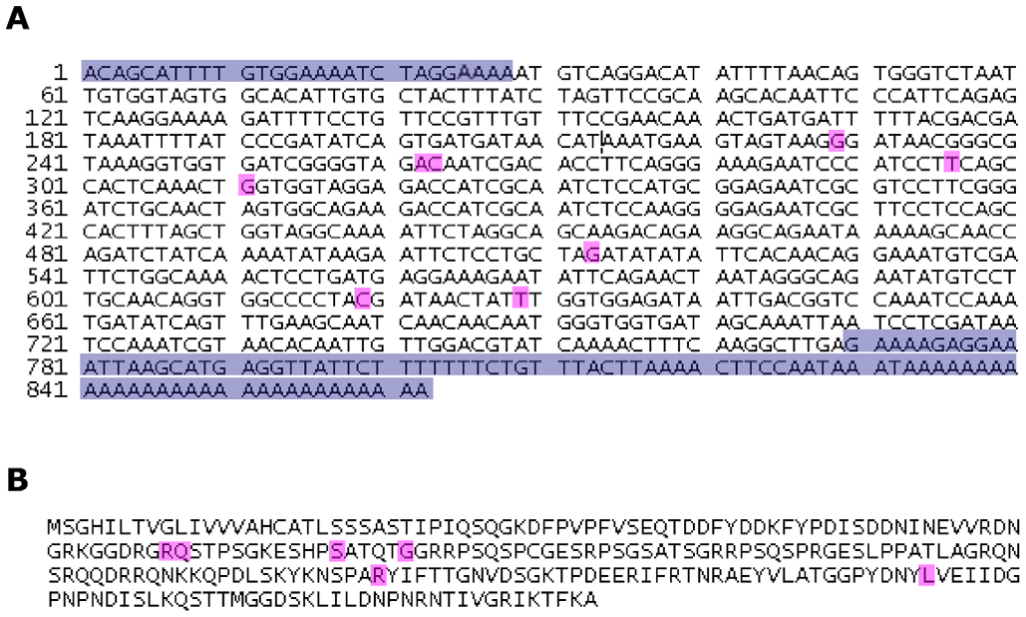
Sequences of the Tunisian *Phlebotomus papatasi* 32 kDa salivary protein. (**A**) mRNA sequence of PpSP32. (**B**) Protein sequence of PpSP32. Differences in the nucleotide and protein sequence PpSP30 described in the GenBank (GenBank AF335490.1) are highlighted in pink. Non coding sequence is highlighted in purple.

**Figure 3:**
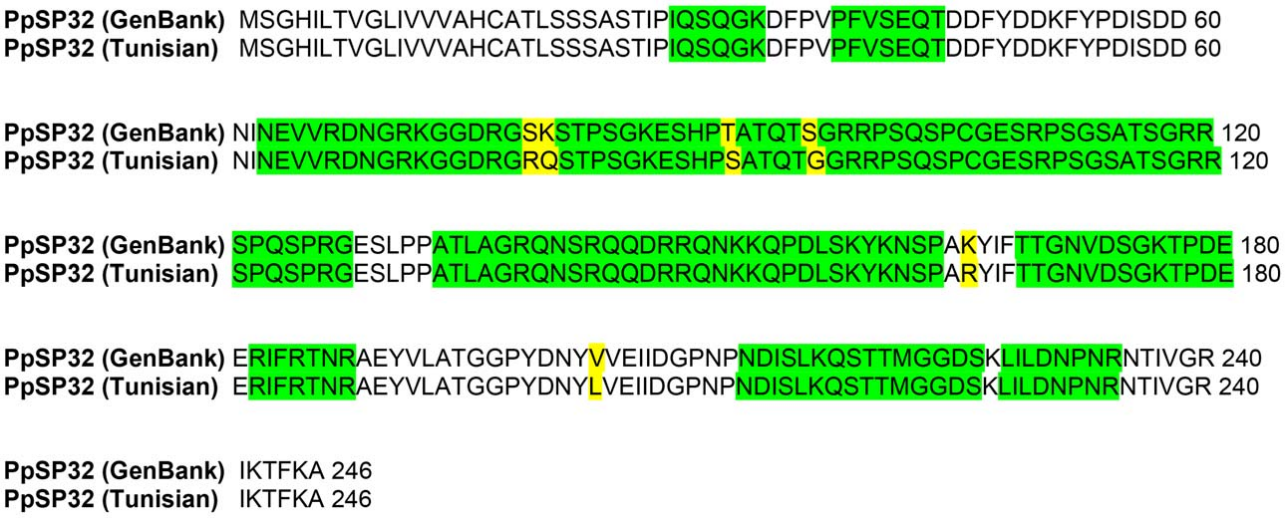
Comparison of predicted B cell epitope between PpSP32 (GenBank AF335490.1) and PpSP32 (Tunisian strain). Yellow shaded amino acids indicate lack of identity. Green shaded amino acids indicate a strong B cell epitope predicted by the bcepred software.

Our preliminary results identified the immunodominant salivary protein of *Phlebotomus papatasi* as a protein of approximately 30 kDa. We thus cloned and sequenced two salivary proteins PpSP30 (Accession number: JX411943) and PpSP32 (Accession number: JX411944) whose predicted molecular weight is around 30 kDa. Our results showed that the Tunisian PpSP30 transcript exhibited five differences within the nucleotide sequence compared to PpSP30 described in GenBank (AF335489.1) (**Figure 1A**) resulting in only one amino acid difference of PpSP30 (Leu23Ile) (**Figure 1B**). Regarding the PpSP32, multiple sequence alignments showed that the Tunisian transcript shares 97% sequence identity and 99% similarity with PpSP32 (GenBank AF335490.1). Accordingly, eight differences were noted in the nucleotide sequence between the Tunisian and GenBank PpSP32 which corresponded to only six differences in the amino acid sequence of the protein (**Figure 2A and 2B**). The predicted secondary structure composition of PpSP32 showed slight differences in the percentage of secondary structure between the two proteins (**Table 1**). As shown in **Table 2**, the Tunisian PpSP32 displays only one difference in a putative motif of post-translational modifications compared to PpSP32 of GenBank, which corresponds to the loss of a PKC phosphorylation site (SGR at position 95). To test whether such differences could be important regarding the antibody recognition, we performed an *in silico* analysis using bcepred software and showed that most of the predictive B cell epitopes are highly conserved between the Tunisian SP32 and the GenBank molecule (**Figure 3**).

**Table 1:** Percentage of secondary structure the Tunisian PpSP32 compared to PpSP32 from Genbank.

Secondary structure Type	Helix	Strand	Loop
% in protein PpSP32 (Tun)	0	19.92	80.08
% in protein PpSP32 (GenBank)	0	21.54	78.46

Tun: Tunisian strain.

**Table 2:** Putative post-translational modification sites present in the amino acid sequence of Tunisian PpSP32 versus the protein described in GenBank.

PKC- Phospho-site		MYRISTYL		AMIDATION	
Tun	GB	Tun	GB	Tun	GB
83 SGK	83 SGK	73 GGDRGR	73 GGDRGS	69 NGRK	69 NGRK
-	95 SGR	111 GSATSG	111 GSATSG	95 GGRR	95 SGRR
115SGR	115SGR	168 GNWDSG	168 GNWDSG	115 SGRR	115 SGRR
122SPR	122SPR				
172SGK	172SGK				
184TNR	184TNR				
214SLK	214SLK				
243 TFJ	243TFJ				

Tun: Tunisian strain; GB: GenBank. PredictProtein software was used for *in silico* structural analysis. Differences are highlighted in bold.

### Serum recognition of the recombinant forms of the Tunisian PpSP30 and PpSP32

**Figure 4:**
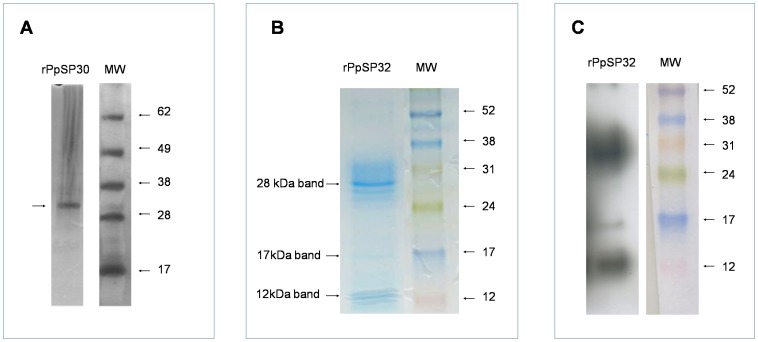
Staining of the recombinant proteins. (**A**) Silver-stained SDS-PAGE gel of recombinant protein rPpSP30. (**B**) Commassie blue-stained SDS-PAGE gel of recombinant protein rPpSP32. (**C**) Western blot analysis of rPpSP32 using monoclonal anti-polyhistidine antibody.

**Figure 5:**
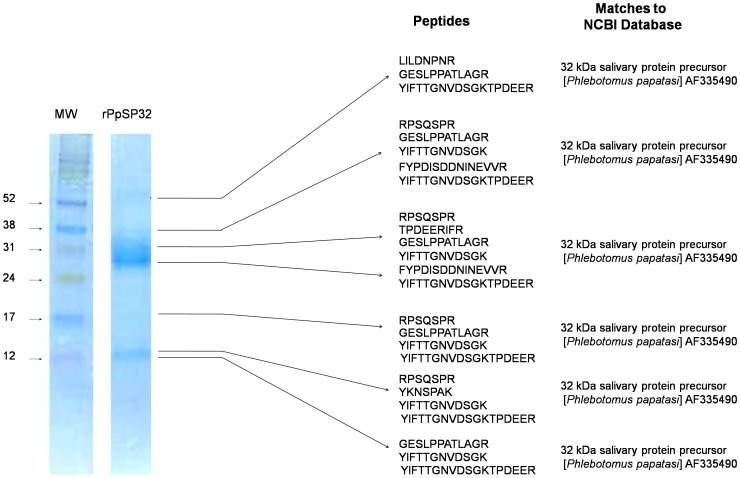
Identification of the different rPpSP32 bands by mass spectrometry. Recombinant PpSP32 (6 µg/well) was run on a 15% SDS- PAGE gel. Seven bands were cut from the gel after staining with Coomassie-blue and mass spectrometry was performed. The RPN800E molecular weight marker (Amersham) was used.

We produced the recombinant proteins PpSP30 and PpSP32 from the Tunisian strain of *Phlebotomus papatasi* in mammalian cells (HEK-293F). These proteins were in solution and did not precipitate or form inclusion bodies-like aggregates. The soluble recombinant proteins were purified with HPLC using a HiTRAP chelating HP column and a second step molecular sieving column to remove imidazole and salt ([17] with modifications). Staining of SDS-PAGE gel demonstrated a unique band of the recombinant PpSP30 (rPpSP30) (**Figure 4A**) while purified recombinant PpSP32 (rPpSP32) showed several bands with a prominent one at a weight of approximately 28 kDa (**Figure 4B**). Mass spectrometry analysis confirmed that all the latter bands are related to PpSP32 and do not correspond to contamination products (**Figure 5**). The presence of different forms of PpSP32 (presence of several bands) was also observed using anti-polyhistidine antibody (**Figure 4C**).

**Figure 6:**
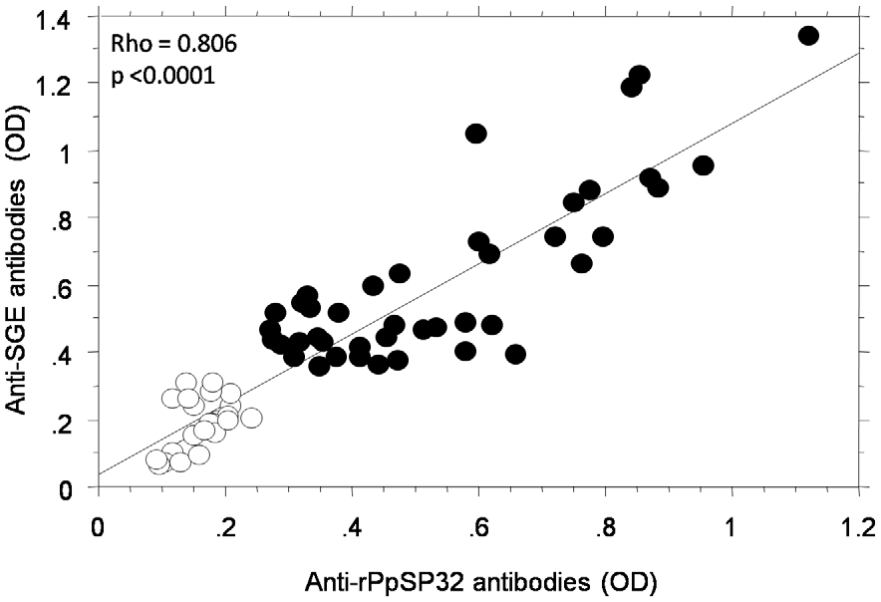
Correlation between the ELISA tests using rPpSP32 and total salivary extract. Twenty-four negative (white circles) and forty-two positive sera (black circles) from donors living in areas with high prevalence of *P. papatasi* previously tested with the total salivary gland extract (SGE) were included.

To test the specific recognition of the recombinant proteins by sera of individuals pre-exposed to *P. papatasi* bites, we performed ELISA and Western blot analyses and utilized a set of serum samples of people living in endemic areas of ZCL (regions of El Guettar and Souk Ejjdid) previously tested against total extract of salivary protein (42 positive sera and 24 negative sera). A positive correlation was found between the OD obtained with rPpSP32 and those obtained with the total extract of salivary gland **(p<0.0001)** (**Figure 6**). No reaction was found with rPpSP30 (**Figure S1**). Comparable results were obtained with sera from another endemic region of ZCL in Tunisia (region of Kairouan). Consistently, 64% of randomly selected donors exhibited specific antibody against rPpSP32 with an excellent correlation between optical densities obtained with this protein and those obtained with the total extract of salivary gland (p<0.0001) **(data not shown)**. Interestingly, we further showed that 18 out of 34 sera tested positive for IgG anti-PpSP32 (52% of cases) exhibited IgE-anti PpSP32 by ELISA **(data not shown)**, a result similar to what previously described when using total salivary extract [13] and suggesting that PpSp32 being the major antigen is equivalent to total salivary extract.

**Figure 7:**
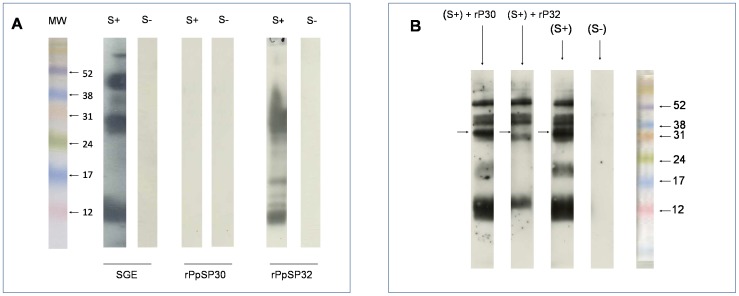
Western blot analyses of native and recombinant salivary proteins. (**A**) Salivary gland extract (SGE) as well as the recombinant forms of PpSP30 (rPpSP30) and PpSP32 (rPpSP32) were run on a 15% SDS-PAGE gel. Western blot analysis was performed with positive (S+) and negative (S−) human sera from donors living in areas with high prevalence of *P. papatasi*. Results are representative of three independent experiments. (**B**) Sera tested positive with IgG anti-SGE were pre-incubated with the recombinant proteins PpSP32 and/or rPpSP30 at 10 µg/ml and then tested in Western blot against SGE. Results are representative of three independent experiments. The arrows indicate the emplacement of the immunodominant protein.

Western blot analyses confirmed the recognition of rPpSP32 by positive sera and revealed several stained bands (**Figure 7A**), a pattern similar to the one obtained with anti-polyhistidine antibody. To further confirm the specific recognition of PpSP32, a competition assay was performed where the human sera where pre-incubated with rPpSP32 or rPpSp30 and tested if they can still recognize the native SP32. As shown in **Figure 7B**, when sera were pre-incubated with the recombinant form of PpSP32, staining of the dominant protein from the total salivary extract disappeared as well as the other potential bands of the native PpSP32 of approximately 12 kDa and 20 kDa (**Figure 7B**). These data suggest that the native protein behaves as the recombinant protein displaying different molecular weights forms and that people exposed to sand flies recognize the different forms of the PpSP32. No differences were seen when PpSP30 was pre-incubated with the sera (**Figure 7B**). This further confirms that the immunodominant protein is PpSP32.

A competition assay was also performed using ELISA test and showed that levels of antibodies directed against total salivary extract was reduced by approximately 50% when sera were pre-incubated with rPpSP32 (**Figure S2**).

**Figure 8:**
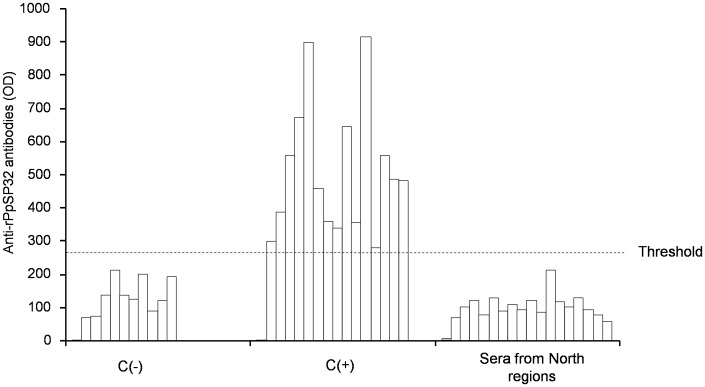
Specificity of rPpSP32 ELISA test towards *P. papatasi* species. Twenty sera obtained from donors living in the North regions of Tunisia where *P. pernicious* is prevalent and *P. papatasi* is absent were tested by ELISA using rPpSP32. Ten negative sera (C−) and fifteen positive sera (C+) from donors living in the regions where *P. papatasi* is prevalent were also included. The threshold of positivity was the mean optical density (OD) of negative controls plus 3 standard deviations.

Finally, to test if rPpSP32 is specific for *P. papatasi* species, we used sera obtained from donors living in the North regions of Tunisia where *P. pernicious* is prevalent and *P. papatasi* is almost absent. None of these sera reacted with rPpSP32 or with the salivary extract of *P. papatasi* (**Figure 8**).

Altogether, our findings indicate that PpSP32 was the dominant target of the antibody response to *P. papatasi* salivary proteins in humans and that the recombinant form of the protein represents a good candidate for large scale testing of human exposure to *P. papatasi* bites.

### Mice immunized with DNA coding to *P. papatasi* salivary proteins confirms immunodominance of PpSP32

**Figure 9:**
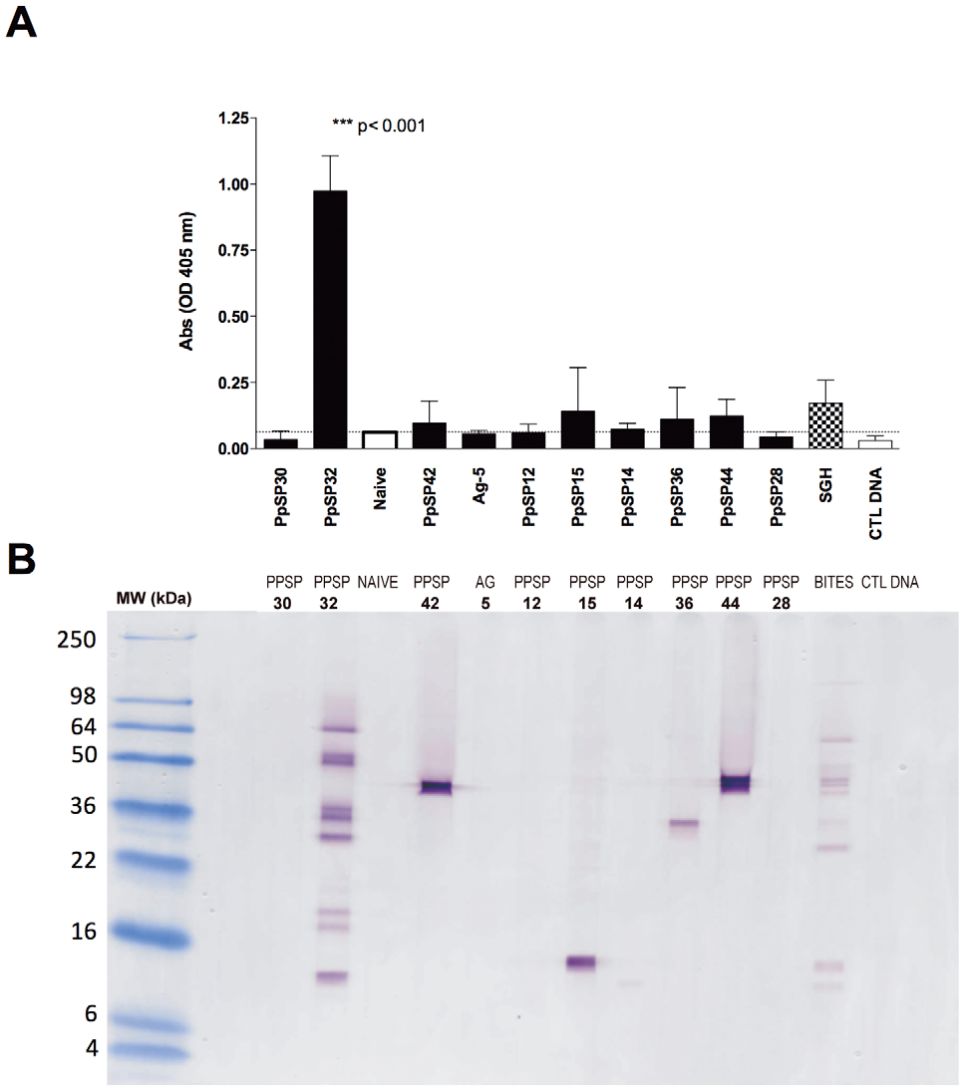
ELISA and Western blot of sera of mice vaccinated with DNA plasmids. (**A**) C57BL/6 mice were immunized three times at two weeks interval with their DNA plasmids coding for the most abundant *P. papatasi* salivary proteins and sera obtained 2 weeks after last immunization. Total anti-saliva IgG was measured by ELISA. Five mice were used in each group. Data are representative of three independent experiments. Bars represent mean ± SD. (**B**) Western blot showing recognition of *P. papatasi* salivary proteins by sera of mice injected with different DNA plasmid coding for *P. papatasi* salivary proteins. Mice sera of each group of plasmids were pooled before testing.

In order to confirm the immunodominance of the PpSP32 experimentally, we immunized mice with DNA coding to PpSP12, PpSP14, PpSP15, PpSP28, Ag-5, PpSP30, PpSP32, PpSP36, PpSP42, PpSP44, SGE and an empty-vector (VR2001). Mice immunized with PpSP14, PpSP15, PpSP32, PpSP36, PpSp42, PpSP44 and SGE presented with measurable levels of anti-saliva IgG antibodies above the anti-saliva ELISA cut-off (**Figure 9A**). As observed in positive human sera from a ZCL endemic area, PpSP32-vaccination induced the highest level of antibodies compared to the immunization with the other *P. papatasi* salivary candidates. Moreover, PpSP30-immunization did not induce detectable levels of IgG antibodies (**Figure 9A**). Mirroring the data observed in humans, the antibodies generated by the PpSP32 DNA-immunization revealed a unique recognition pattern of salivary proteins in a wide range of molecular weights, in contrast to the highly specific antibodies produced by the PpSP42-, PpSp15-, PpSP14-, PpSp36- and PpSP44-vaccination (**Figure 9B**). Interestingly, the pattern of recognition by the PpSP32-immunized mice differs from mice exposed to uninfected *P. papatasi* bites (**Figure 9B**).

## Discussion

In different vector-host models, exposure to vector bites induces the production of specific antibodies and the level of such antibodies has been frequently correlated to the risk of disease development [7]–[12]. Thus, the detection of antibodies against the saliva of hematophagous insect vectors could be used as an indicator of vector exposure and in some instances as an indicator for risk of contracting disease. However, performing large-scale serological studies to detect vector exposure using total salivary gland extracts is limited by the difficulty to standardize such assays due to the potential variability in stocks of salivary gland extracts. The use of recombinant protein may reduce such a problem. Herein, we identified PpSP32 as the immunodominant target of the antibody response to salivary proteins of *P. papatasi* and used the recombinant form of the protein in a serological test as a biomarker of exposure to sand fly bites in Tunisia

Sand fly saliva is known to contain a variety of compounds that are highly immunogenic. Antibodies against saliva of sand flies have recently been evaluated as potential tools for measurement of exposure to sand fly bites [18], [19]. Current data provide evidence that the levels of specific antibodies correlate with variations in vector exposure [18] and could, thus, be used to evaluate vector control intervention [20]. Moreover, in endemic areas for cutaneous leishmaniasis, the presence of anti-saliva antibodies correlated with the risk of contracting disease [4], [7]. Consistently, we have recently provided data supporting the use of *P. papatasi* saliva antibodies as biomarker to evaluate exposure to the fly in endemic areas of ZCL in Tunisia [13]. In fact, nearly 90% of children who tested negative for IgG antibodies against *P. papatasi* saliva developed specific antibodies after two transmission seasons of *L. major* [13]. Interestingly, children with high titer of anti-saliva antibodies were more likely to develop ZCL [13]. Nevertheless, it would be expected that technical challenges in obtaining standardized salivary gland extracts may hampers the use of such ELISA tests. Using recombinant proteins could be a promising approach in screening human exposure to the sand fly bites [19], [21] and perhaps in evaluating the risk of developing the disease. We thus focused our work on characterizing the immunodominant salivary protein of *P. papatasi*, the vector of ZCL in Central Tunisia and producing the related recombinant protein. Testing such recombinant protein would validate its usefulness as a specific biomarker of *P. papatasi* exposure in humans. All positive sera from naturally exposed children reacted strongly in Western blot with a band of approximately 30 kDa [13]. Previous mass spectrometry revealed that this band may probably correspond to PpSP30. Yet, such data needed further confirmation since other proteins could be present in that mix like PpSP32 that runs at a molecular weight close to 30 kDa and could have not been detected by this technique due to amount of protein present in the salivary gland preparation. We thus cloned and sequenced the two salivary proteins from the Tunisian strain of *P. papatasi* with a molecular weight close to 30 kDa, namely PpSP30 and PpSP32.

Previous studies showed that the composition and antigenicity of sand fly saliva may vary with the geographical origin of the fly [22]–[25]. Accordingly, our results showed that the PpSP30 from the Tunisian strain of *P. papatasi* exhibited five differences within the nucleotide sequence compared to the strain of PpSP30 described in GenBank. These differences resulted in one difference in the protein sequence of PpSP30 (Leu23Ile). For PpSP32, the immunodominant protein, we observed a high level of identity (98%) between the tunisian strain and the related protein from GenBank. Accordingly, only eight differences were noted in the nucleotide sequence, which correspond to six differences in the protein sequence. Furthermore, a B cell epitope prediction analysis suggested that at least six epitopes between these two sequences are 100% identical suggesting that PpSP32 could be of potential use as marker of exposure in different geographical areas. Expression of the salivary proteins in mammalian expression system produced these proteins in the soluble form that probably will be close to the folding form of the native protein. While staining of SDS PAGE gel showed a unique band of the recombinant rPpSP30, it showed several bands for the recombinant rPpSP32. All these different forms are related to PpSP32 as confirmed by mass spectrometry and may possibly correspond to different conformations of this protein in solution and different glycosylated forms of the protein. The larger than 30 kDa forms may be explained by glycosylations or other post-translational modifications. The smaller molecular forms, however, could be the result of degradation (which may probably not be the case because these forms were observed in native proteins when DNA plasmid of SP32 was used to immunize mice) or to different conformations of the proteins that will not permit sodium dodecyl sulfate (SDS) to open up the protein and will give the protein the appearance to run at apparent smaller molecular weights. This possibility is supported by amino acid composition of PpSP32 which is rich in glycine and serine and a significant number of glutamine. Interestingly, the Western-blot using anti-polyhistidine antibody revealed the same pattern further suggesting that the different bands were not related to degradation products but could correspond to different molecular forms.

We further showed that recombinant PpSP32 was strongly recognized by human sera. This suggests that SP32 salivary protein from *P. papatasi* is the immunodominant one and that the related recombinant form exhibits epitopes that are recognized by antibodies from donors immunized with the native PpSP32. In a previous report showing that all positive sera from naturally exposed children reacted strongly with a band of approximately 30 kDa, mass spectrometry revealed that this band corresponded probably to PpSP30 [13]. Yet, our current work does not corroborate this hypothesis. This seems to be a good opportunity to point out that the presence of a protein on a single SDS-PAGE does not mean that a particular protein is the only protein present in the band. We recently produced a cDNA library from the salivary glands of *P. papatasi* females (Tunisian strain) and demonstrated that PpSP32 is less represented than the members of D7 family (PpSP30 and PpSP28) (Maha Abdeladhim et al. In press) explaining in part why PpSP32 was not identified in the previous mass spectrometry analysis. N-terminal blockage of the proteins, which will result in a negative result when performing Edman degradation, could be another reason explaining why other proteins are not detected in the mix. Other reason could be that some proteins are not ionized properly due to their amino acid composition (PpSP32 has a high content of serine and glycine) and this could result in a negative result in a mass spectrometry analysis. One of the main objectives of this work was to test the hypothesis that the PpSP30 was the immunogenic protein and the current result refute this hypothesis and provides now strong evidence that SP32 is the immunogenic and immunodominat antigen. Consistently, vaccination of mice with PpSP32 induced the highest levels of antibodies compared to other *P. papatasi* sand fly molecules. Our further experiments showed that the binding of serum antibodies to the native PpSP32 from total salivary extract could be inhibited by addition of the recombinant form of PpSP32 further supporting that recombinant form of PpSP32 is similar to the native protein. Interestingly, the pre-incubation of positive sera with rPpSP32 led to the disappearance of bands of approximately 12 kDa and 20 kDa which may represent the other “native” forms of PpSP32. Accordingly, antibodies generated by PpSP32-immunization of mice recognized several bands of salivary extract of different molecular weights. This rather suggests that the native protein behaves as the recombinant protein displaying different molecular weights and that people exposed to sand flies will recognize the different forms of the PpSP32 protein. Notably, PpSP32 DNA vaccination of mice also induces antibodies recognizing a large number of bands but the pattern and intensity of such bands differ from those found in animals exposed to sand fly bites. We can hypothesize that this may be related to the total amount of protein injected and the total amount for the booster immunization as well. The amount of the protein (SP32) in the glands is not as large as compared to other proteins including SP30. Additionally, this may be due to the higher levels of specific antibodies induced by immunization with the DNA plasmids compared to bites as it can be observed by the intensity of recognized proteins after vaccination with PpSP32, PpSP42, PpSP15, PpSP36 and PpSP44 (Figure 9B).

The complexity of sand fly saliva is such that different species of sand flies contain specific antigens [22], [26]–[29]. For use as an epidemiological tool, the antigen used should be specific to the vector, sensitive enough to measure exposure, and exhibit a low persistence in the host to accurately reflect changes in vector exposure over time. The PpSP32-like family of proteins previously identified in *P. papatasi* salivary glands has only been found in sand flies and their functions are still unknown [26], [30]. PpSP32 seems to be a good candidate for measurement exposure to *P. papatasi* bites. Indeed, it is abundantly present in the saliva of sand flies and is expressed without any influence from the diet received or the age of the fly [31]. PpSP32 is common to at least five different sand flies (*P. papatasi, P. argentipes, P. ariasi, P. perniciosus and L. longipalpis*) [26]. Cross-reactivity between sand fly species, however, may not be an issue since some preliminary experiments suggest the specificity of our serological test towards the SP32 from *P. papatasi*. Indeed, the sera of 20 donors living in the North of Tunisia (regions of Utique and Menzel Bourguiba) where *P. perniciosus* is frequent and *P. papatasi* is absent did not show any reactivity towards the rPpSP32. Additional experiments will help us to define the optimal conditions of the serological test using recombinant PpSP32. The inclusion of individuals from non-endemic areas will help us to establish cut-off values. Different immunoglobulin isotypes (IgG subclasses and IgE isotype) will also be tested in order to verify whether detecting IgG subclasses and/or IgE antibody is more specific to PpSP32 than detecting total IgG antibody. Finally, the assay will be validated on using a large panel of individuals at risk, randomly selected from a new epidemiological survey. The correlation between the levels of antibodies against salivary proteins and those against the recombinant protein will be evaluated. The overall performance of the serology using recombinant protein will be tested by calculating the sensitivity, the specificity, and the positive and negative predictive values as recently described in a serological test performed to probe for vector exposure to another sand fly, *Lutzomya longipalpis* [21].

In conclusion, we have identified the immunodominant salivary protein from *P. papatasi* that is targeted by serum antibodies from humans living in an endemic area of ZCL and demonstrated the suitability of using the recombinant form of this protein in a serological test. Once tested on a wider scale, this test could become a promising epidemiological tool for accurate surveillance of exposure to the vector of ZCL in Tunisia. Such test also could be useful to monitor the emergence of new foci of ZCL as well as to evaluate the efficiency of control measures that reduce the contact with the vector. Saliva-based tests would, in fact, be more appropriate to assess the effectiveness of anti-vectorial devices than using entomological methods (i.e., human landing catch) or measuring vector-borne disease incidence [32]. Moreover, the existence of a correlation between anti-saliva antibodies and the risk of leishmaniasis underlines the interest of such a test for predicting the risk of leishmaniasis.

## Supporting Information

Figure S1 **ELISA test using recombinant PpSP30.** Twenty-five donors with specific antibodies against total salivary gland extract (SGE) were tested by ELISA using the recombinant protein rPpSP30.
(TIF)

Figure S2 **Competition assay using ELISA test.** Ten sera from donors with specific antibodies against total salivary gland extract (SGE) were pre-incubated with the recombinant proteins PpSP32 at 10 µg/ml and then tested by ELISA against SGE.
(TIF)
