# Correction: Salivary Antigen SP32 Is the Immunodominant Target of the Antibody Response to *Phlebotomus papatasi* Bites in Humans

**DOI:** 10.1371/journal.pntd.0012303

**Published:** 2024-07-03

**Authors:** Soumaya Marzouki, Maha Abdeladhim, Chaouki Ben Abdessalem, Fabiano Oliveira, Beya Ferjani, Dana Gilmore, Hechmi Louzir, Jesus G. Valenzuela, Mélika Ben Ahmed

The rPpSP30 panel in Fig 4A was beautified to remove a non-specific band between 17kDa and 28kDa that was present on the original gel. The image in Fig 4B was spliced to remove irrelevant lanes. Fig 4 is corrected to replace the rPpSP30 panel in Fig 4A with the unedited image and to indicate the splice location in Fig 1B. The original images underlying this figure are provided in S1 File.

**Fig 4:**
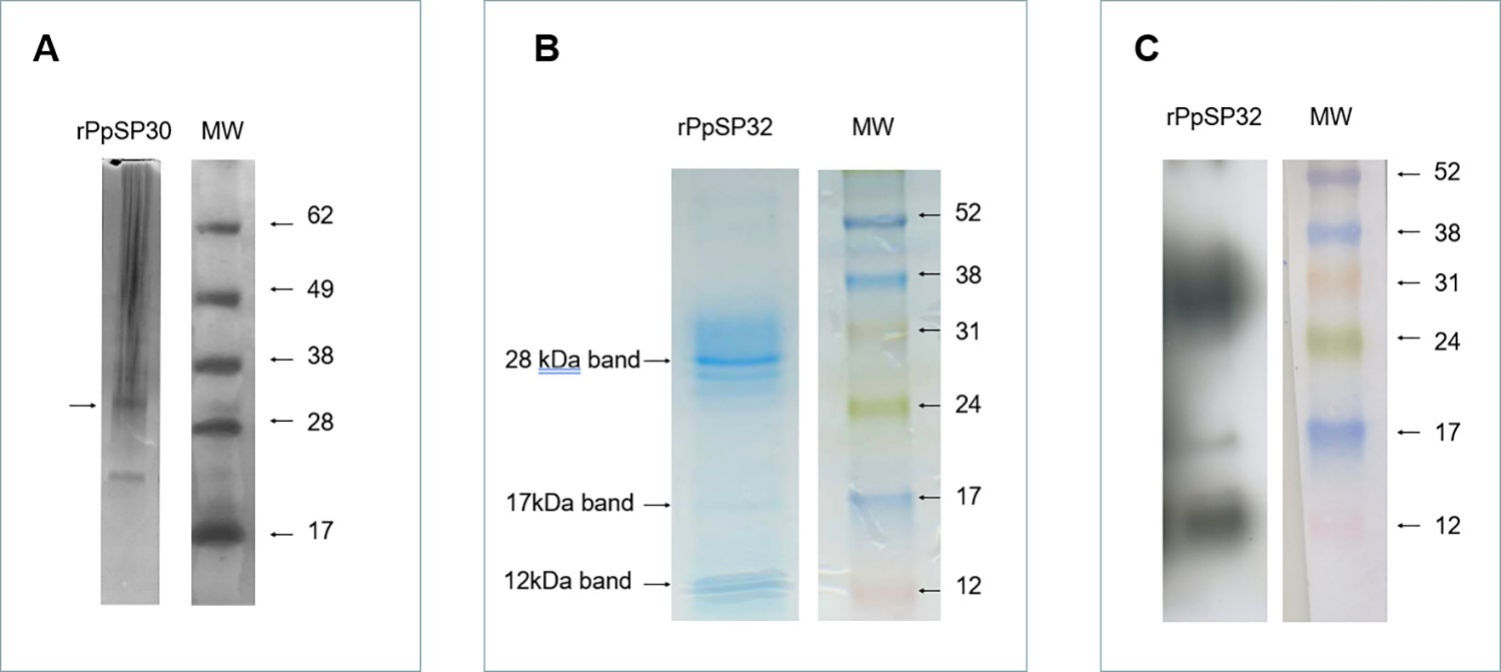
Staining of the recombinant proteins. (A) Silver-stained SDS-PAGE gel of recombinant protein rPpSP30. (B) Coomassie blue-stained SDS-PAGE gel of recombinant protein rPpSP32. (C) Western blot analysis of rPpSP32 using monoclonal anti-polyhistidine antibody.

In Fig 7A, the S+ and S- rPpSP30 panels were erroneously duplicated and the SGE lanes originated from a different blot. Fig 7A is corrected here to resolve the duplication and to replace the SGE S+ lane with data measured on the same blot. SGE S- was measured on different blots, provided in S2 File with the underlying images for Fig 7.

**Fig 7:**
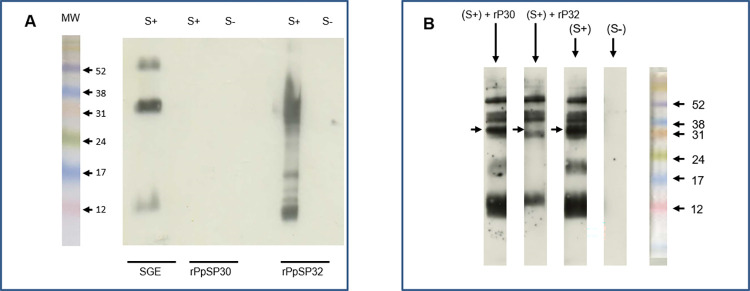
Western blot analyses of native and recombinant salivary proteins. (A) Salivary gland extract (SGE) as well as the recombinant forms of PpSP30 (rPpSP30) and PpSP32 (rPpSP32) were run on a 15% SDS-PAGE gel. Western blot analysis was performed with positive (S+) and negative (S−) human sera from donors living in areas with high prevalence of *P*. *papatasi*. Results are representative of three independent experiments. There is a blank lane between rPpSP30 S- and rPpSP32 S+. (B) Sera tested positive with IgG anti-SGE were pre-incubated with the recombinant proteins PpSP32 and/or rPpSP30 at 10 µg/ml and then tested in Western blot against SGE. Results are representative of three independent experiments. The arrows indicate the emplacement of the immunodominant protein.

In the preparation of Fig 7B, the image was spliced for presentation and to remove an unnecessary lane. The original underlying image is provided in S2 File.

The original underlying data to support all other results in the article and Supporting Information files are available from the corresponding author.

The authors apologize for the errors in the published article.

## Supporting information

S1 File Original images underlying Fig 4. (PPTX)

S2 File Original images underlying Fig 7. (PPTX)
