# Supplementary figures and images for: Salivary Antigen SP32 Is the Immunodominant Target of the Antibody Response to Phlebotomus papatasi Bites in Humans
Source: PLoS Negl Trop Dis. 2012 Nov 29;6(11):e1911. doi: 10.1371/journal.pntd.0001911 (PMC3510156; doi:10.1371/journal.pntd.0001911)

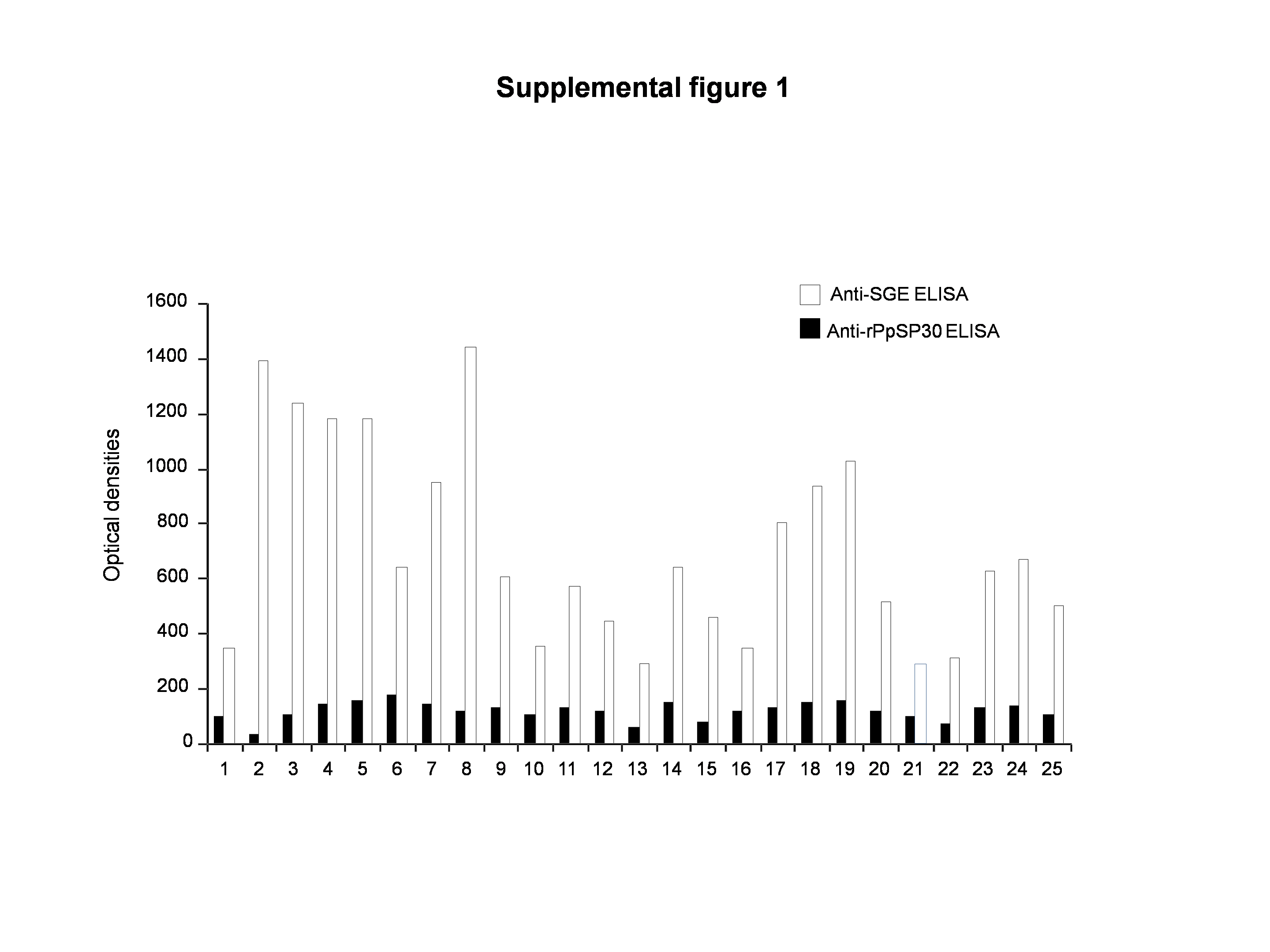

Supplement: Figure S1 — ELISA test using recombinant PpSP30. Twenty-five donors with specific antibodies against total salivary gland extract (SGE) were tested by ELISA using the recombinant protein rPpSP30. (TIF) [file pntd.0001911.s001.tif]

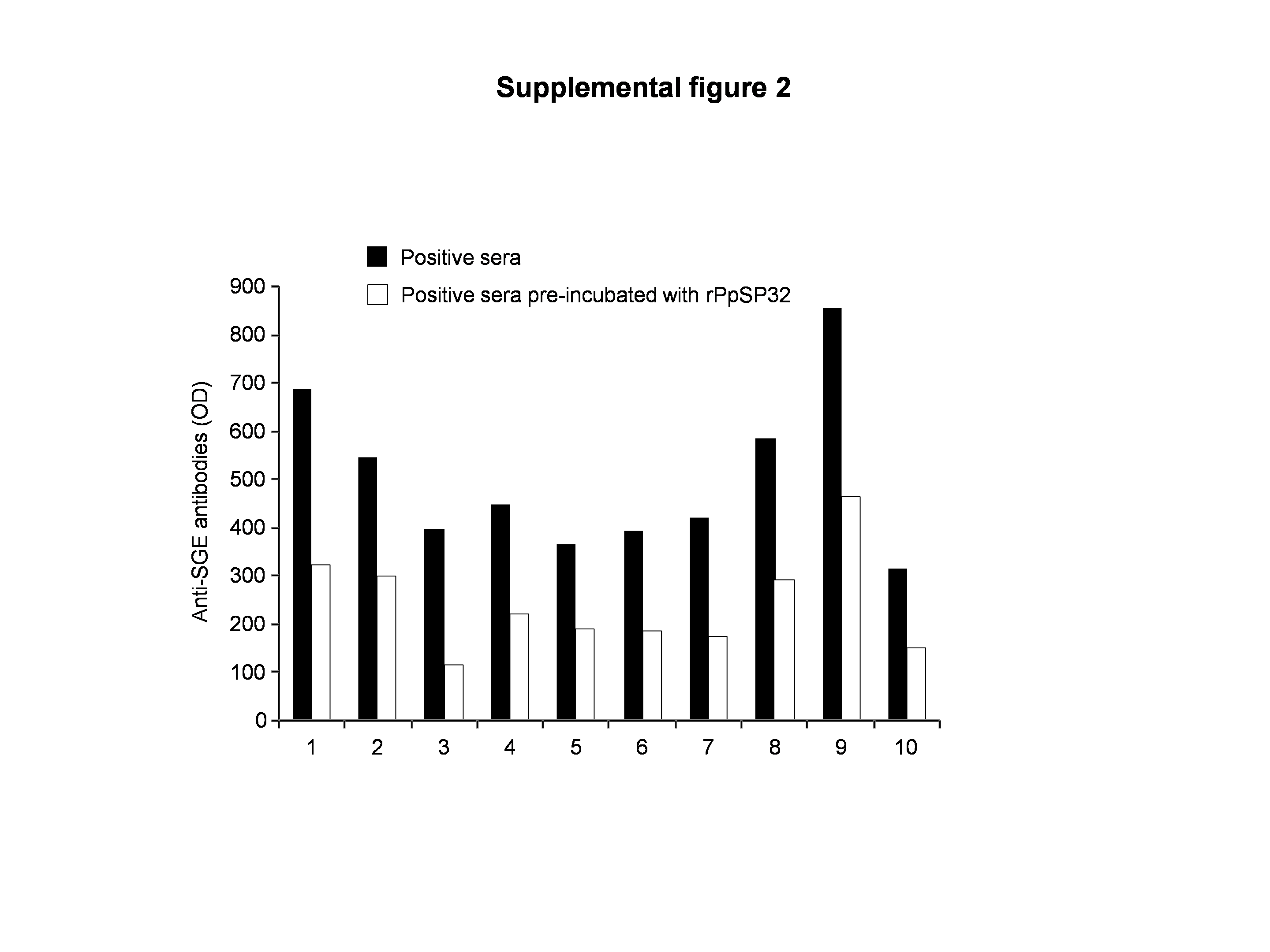

Supplement: Figure S2 — Competition assay using ELISA test. Ten sera from donors with specific antibodies against total salivary gland extract (SGE) were pre-incubated with the recombinant proteins PpSP32 at 10 µg/ml and then tested by ELISA against SGE. (TIF) [file pntd.0001911.s002.tif]
