# Supplementary material for: Correction: Salivary Antigen SP32 Is the Immunodominant Target of the Antibody Response to Phlebotomus papatasi Bites in Humans
Source: PLoS Negl Trop Dis. 2024 Jul 3;18(7):e0012303. doi: 10.1371/journal.pntd.0012303 (PMC11221742; doi:10.1371/journal.pntd.0012303)
Supplement: S1 File — (PPTX) [file pntd.0012303.s001.pptx]

## Slide 1
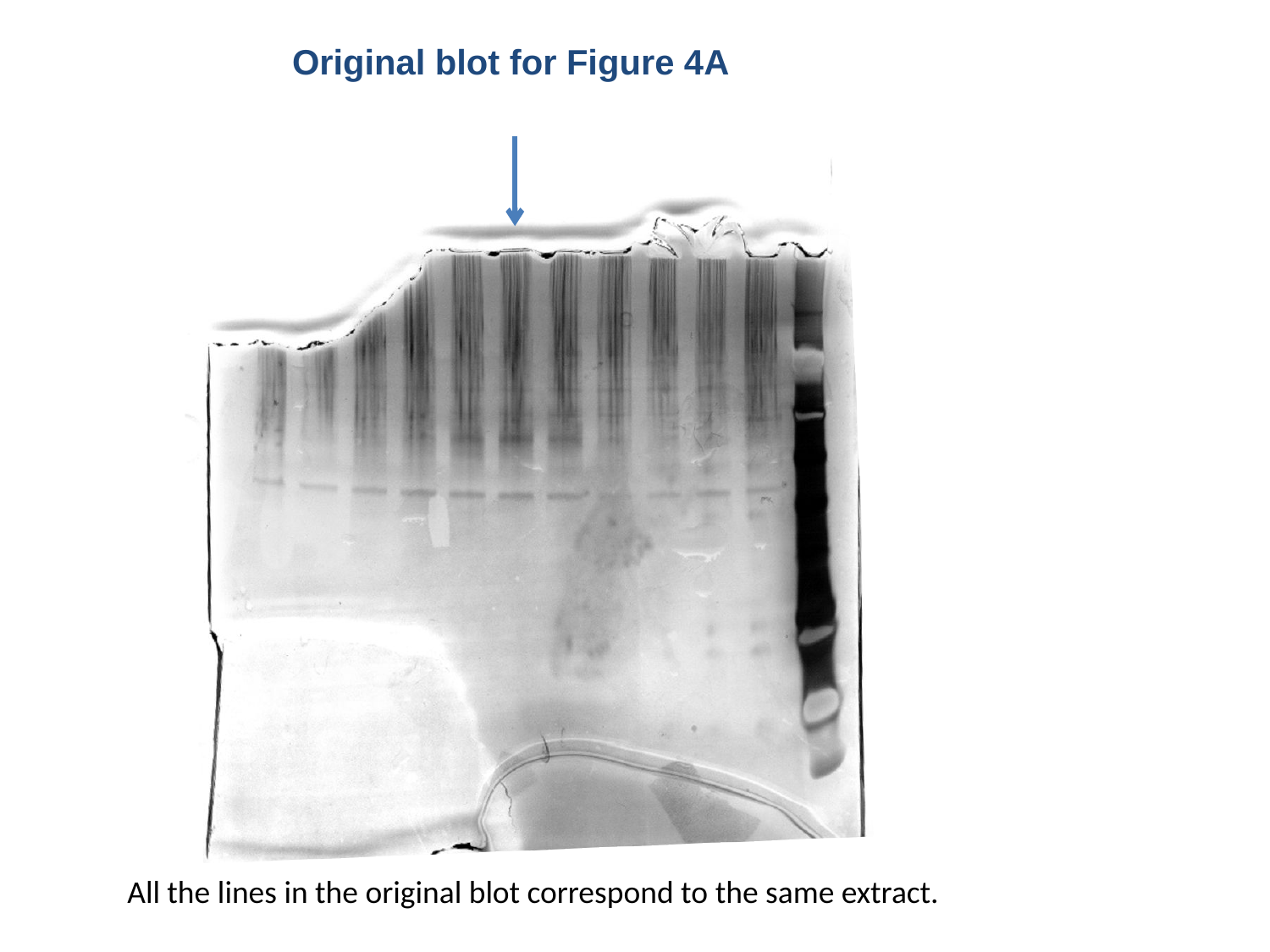

Original blot for Figure 4A
All the lines in the original blot correspond to the same extract.

## Slide 2
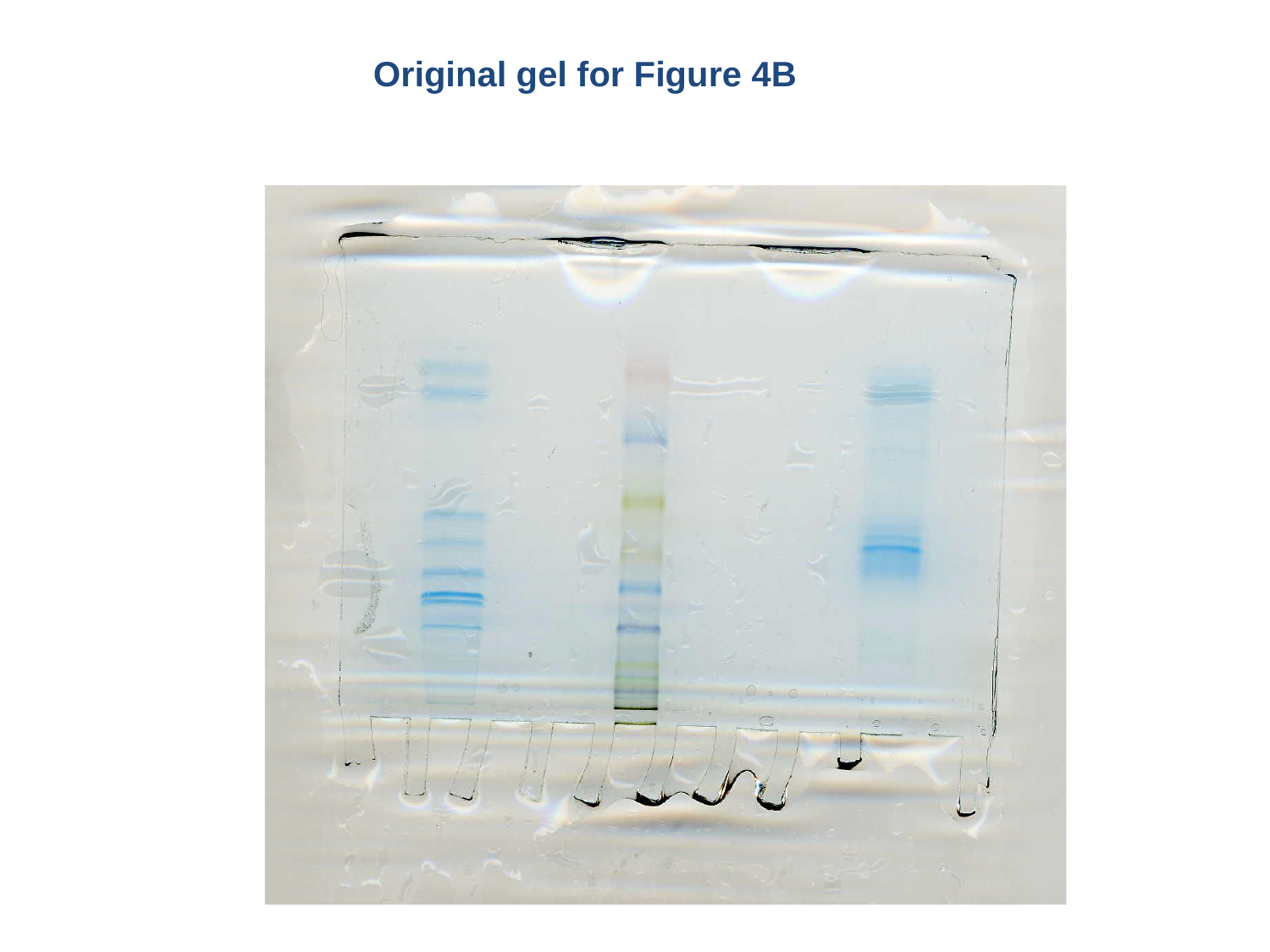

Original gel for Figure 4B

## Slide 3
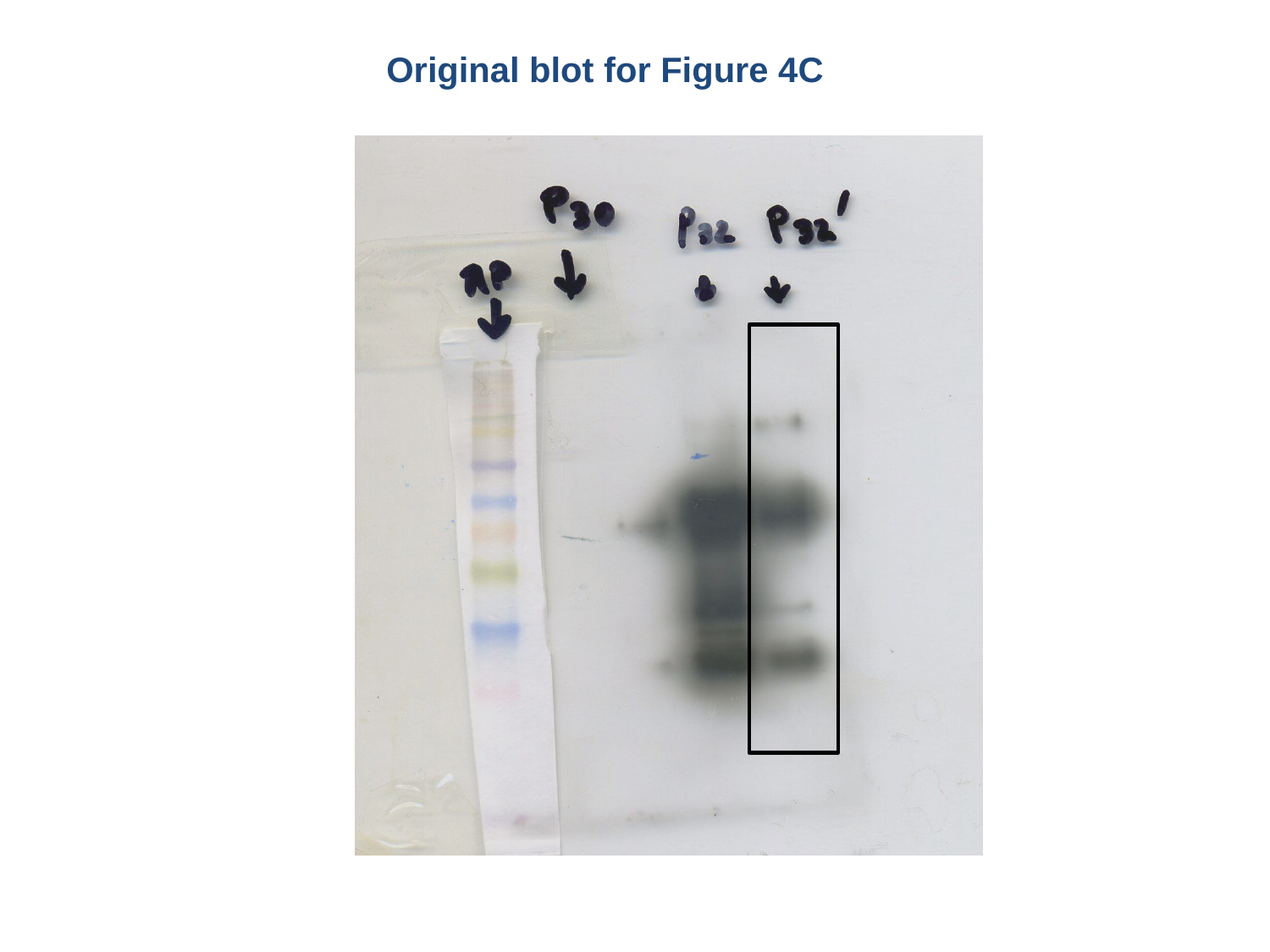

Original blot for Figure 4C
