# Supplementary material for: Correction: Salivary Antigen SP32 Is the Immunodominant Target of the Antibody Response to Phlebotomus papatasi Bites in Humans
Source: PLoS Negl Trop Dis. 2024 Jul 3;18(7):e0012303. doi: 10.1371/journal.pntd.0012303 (PMC11221742; doi:10.1371/journal.pntd.0012303)
Supplement: S2 File — (PPTX) [file pntd.0012303.s002.pptx]

## Slide 1
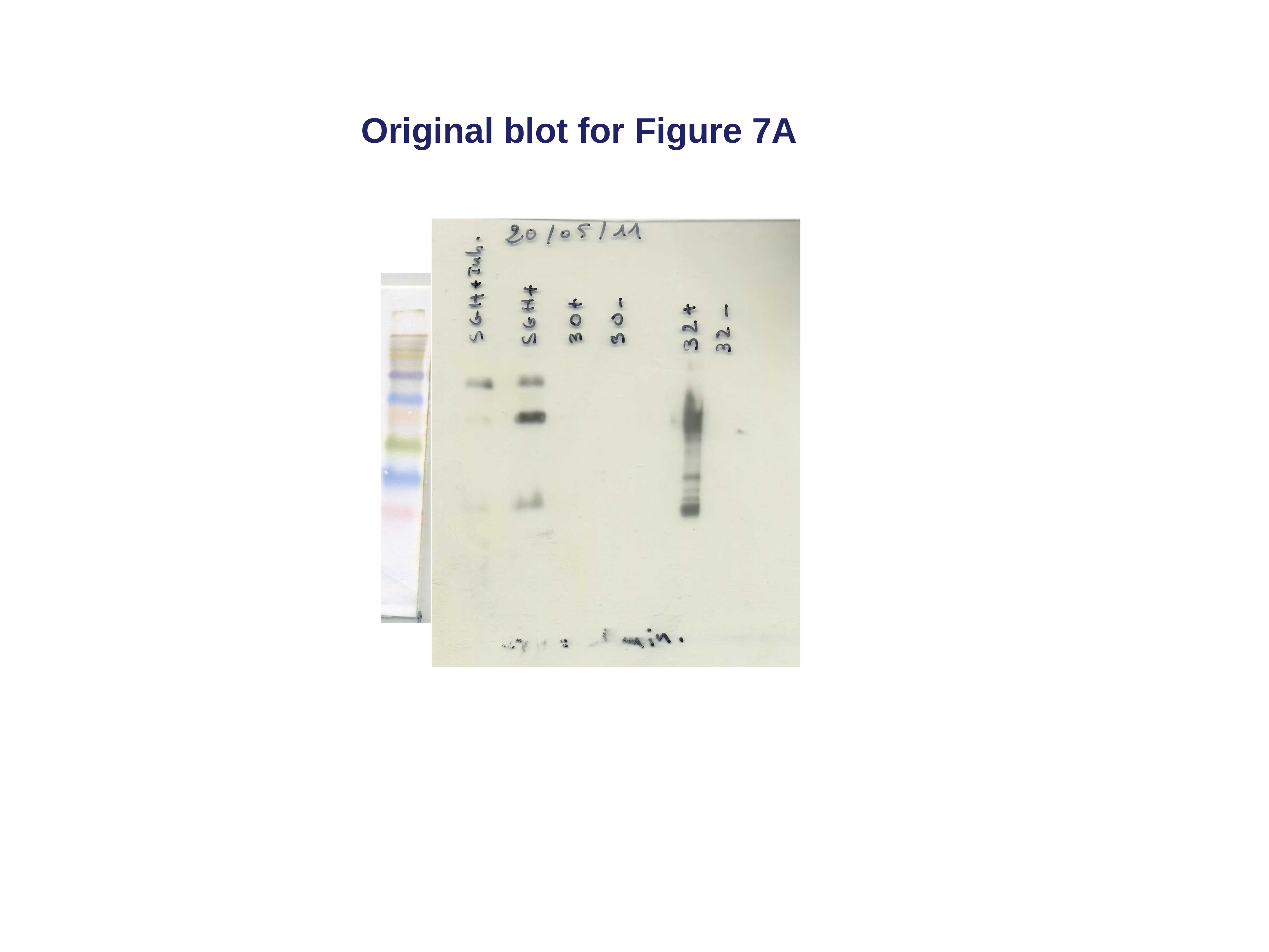

Original blot for Figure 7A

## Slide 2
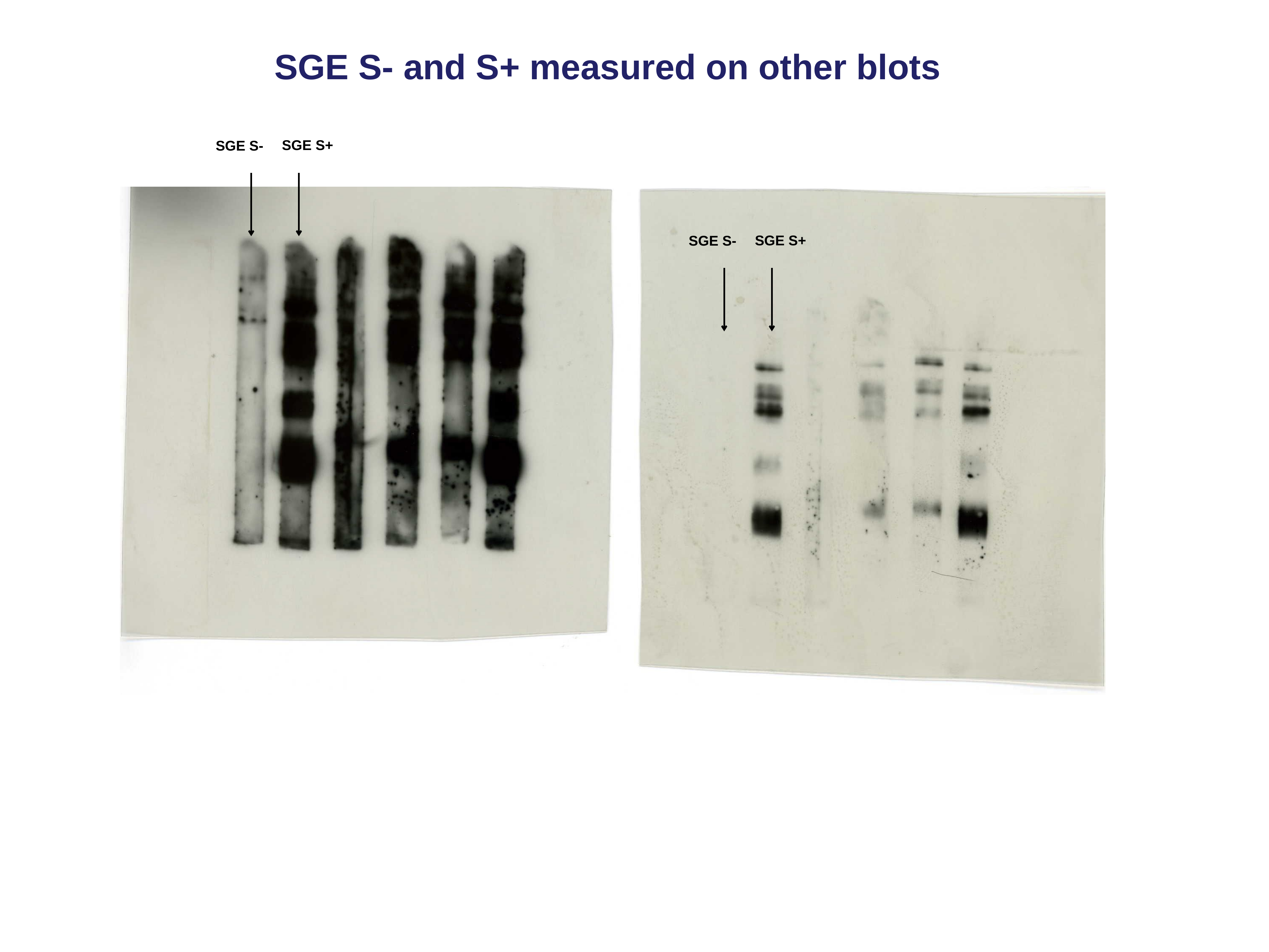

SGE S- and S+ measured on other blots
SGE S+
SGE S-
SGE S+
SGE S-

## Slide 3
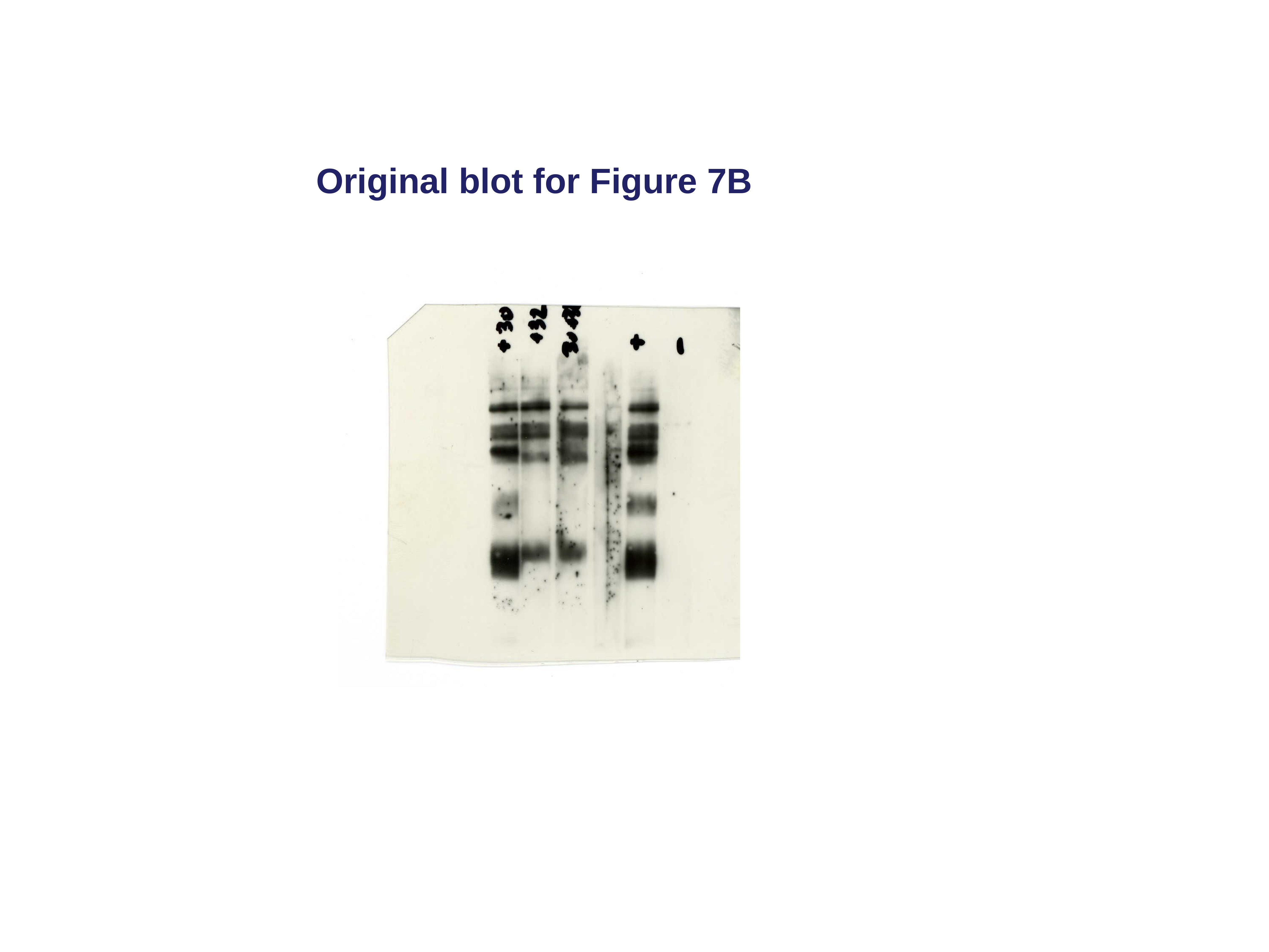

Original blot for Figure 7B
